# Antibodies against Serum Anti-Melanoma Differentiation-Associated Gene 5 in Rheumatoid Arthritis Patients with Chronic Lung Diseases

**DOI:** 10.3390/medicina59020363

**Published:** 2023-02-14

**Authors:** Shomi Oka, Takashi Higuchi, Hiroshi Furukawa, Kota Shimada, Akira Okamoto, Atsushi Hashimoto, Akiko Komiya, Koichiro Saisho, Norie Yoshikawa, Masao Katayama, Toshihiro Matsui, Naoshi Fukui, Kiyoshi Migita, Shigeto Tohma

**Affiliations:** 1 Department of Rheumatology, National Hospital Organization Tokyo National Hospital, 3-1-1 Takeoka, Kiyose 204-8585, Japan; 2 Clinical Research Center for Allergy and Rheumatology, National Hospital Organization Sagamihara National Hospital, 18-1 Sakuradai, Minami-ku, Sagamihara 252-0392, Japan; 3 Department of Nephrology, Ushiku Aiwa General Hospital, 896 Shishiko-cho, Ushiku 300-1296, Japan; 4 Department of Rheumatology, National Hospital Organization Sagamihara National Hospital, 18-1 Sakuradai, Minami-ku, Sagamihara 252-0392, Japan; 5 Department of Rheumatic Diseases, Tokyo Metropolitan Tama Medical Center, 2-8-29 Musashi-dai, Fuchu 183-8524, Japan; 6 Department of Rheumatology, National Hospital Organization Himeji Medical Center, 68 Hon-machi, Himeji 670-8520, Japan; 7 Department of Internal Medicine, Sagami Seikyou Hospital, 6-2-11 Sagamiohno, Minami-ku, Sagamihara 252-0303, Japan; 8 Department of Clinical Laboratory, National Hospital Organization Sagamihara National Hospital, 18-1 Sakuradai, Minami-ku, Sagamihara 252-0392, Japan; 9 Department of Orthopedics/Rheumatology, National Hospital Organization Miyakonojo Medical Center, 5033-1 Iwayoshi-cho, Miyakonojo 885-0014, Japan; 10 Tanimura Hospital, 10-2 Kitakoji, Nobeoka 882-0041, Japan; 11 Department of Internal Medicine, National Hospital Organization Nagoya Medical Center, 4-1-1 Sannomaru, Naka-ku, Nagoya 460-0001, Japan; 12 Department of Life Sciences, Graduate School of Arts and Sciences, The University of Tokyo, 3-8-1 Komaba, Meguro-ku, Tokyo 153-8902, Japan; 13 Clinical Research Center, National Hospital Organization Nagasaki Medical Center, 2-1001-1 Kubara, Omura 856-8562, Japan; 14 Department of Gastroenterology and Rheumatology, Fukushima Medical University School of Medicine, 1 Hikarigaoka, Fukushima 960-1295, Japan

**Keywords:** rheumatoid arthritis, anti-melanoma differentiation-associated gene 5 antibodies, airway diseases, chronic lung disease

## Abstract

Chronic lung diseases (CLD), including interstitial lung disease (ILD) and airway diseases (ADs), are common complications of rheumatoid arthritis (RA). Rheumatoid factor (RF) and anti-citrullinated peptide antibodies are reported to be associated with CLD in RA patients. The presence of anti-melanoma differentiation-associated gene 5 (MDA5) antibodies (Abs) is associated with clinically amyopathic dermatomyositis developing into rapidly progressive ILD. However, few studies on anti-MDA5 Abs in RA have been published. Here, we analyzed the association of anti-MDA5 Abs with CLD complications in RA. Anti-MDA5 Abs were quantified in sera from RA patients with or without CLD. Anti-MDA5 Ab levels were higher in RA patients with ADs than without (mean ± SDM, 4.4 ± 2.4 vs. 4.0 ± 4.2, *p =* 0.0001). AUC values of anti-MDA5 Ab and RF ROC curves were similar in RA patients with or without CLD (0.578, 95%CI 0.530–0.627 and 0.579, 95%CI 0.530–0.627, respectively, *p =* 0.9411). Multiple logistic regression analysis of anti-MDA5 Abs and clinical characteristics yielded an MDA5-index with a higher AUC value than anti-MDA5 Ab alone (0.694, 95%CI 0.648–0.740, *p =* 5.08 × 10^−5^). Anti-MDA5 Abs were associated with ADs in RA patients and could represent a biomarker for CLD, similar to RF. The involvement of anti-MDA5 Abs in the pathogenesis of ADs in RA is proposed.

## 1. Introduction

Rheumatoid arthritis (RA) is an autoimmune disease characterized by the destruction of synovial joints. Chronic lung diseases (CLD) are frequently present in RA, and include interstitial lung disease (ILD), airway diseases (ADs) and emphysema. The complication of ILD or ADs confers a dismal prognosis for RA patients [1,2,3,4,5]. Usual interstitial pneumonia (UIP) is especially associated with very poor prognosis in RA patients [6]. It is therefore important to clarify the pathogenesis of ILD and ADs in RA patients.

Krebs von den lungen-6 (KL-6) and surfactant protein-D (SP-D) are biomarkers for idiopathic pulmonary fibrosis, and also for ILD in RA [7,8]. It has also been reported that KL-6 and SP-D are increased in ADs and emphysema [9,10]. Rheumatoid factors (RFs) are antibodies (Abs) against the Fc portion of immunoglobulin G. Anti-citrullinated peptide antibodies (ACPAs) are Abs against citrullinated peptides generated by posttranslational modification of arginine residues. RF and ACPA are used as rheumatoid arthritis classification criteria [11]. RFs are associated with ILD in RA [12,13]. ACPAs are also associated with ILD in RA [12,14,15]. The presence of RF is associated with mortality of RA patients [16]. RF and ACPA are considered to be biomarkers for ILD in RA [17].

Anti-melanoma differentiation-associated gene 5 (MDA5) Abs are directed against RNA helicase. Their presence is associated with clinically amyopathic dermatomyositis developing into rapidly progressive ILD with a poor prognosis [18,19,20,21]. It has been reported that anti-MDA5 Abs are not present in RA patients [22]. However, few validation studies on anti-MDA5 Abs in RA with CLD have been conducted. In the present study, we investigated the association of anti-MDA5 Abs with CLD in RA patients.

## 2. Materials and Methods

### 2.1. Patients

RA patients (*n* = 558) were recruited at Himeji Medical Center, Miyakonojo Medical Center, Nagasaki Medical Center, Nagoya Medical Center, Sagamihara Hospital and Tokyo Hospital. All patients fulfilled the rheumatoid arthritis classification criteria [11], or American College of Rheumatology criteria for RA [23]. They were diagnosed as having UIP, nonspecific interstitial pneumonia (NSIP), ADs, emphysema, or no CLD, based on the predominant findings of chest computed tomography; the findings of ADs are centrilobular or peribronchial nodules, branching linear structures, bronchial dilatation, bronchial wall thickening, or atelectasis [9]. The CLD(+) group includes UIP, NSIP, ADs, and emphysema and ILD groups include UIP and NSIP patients. Sera were collected from these RA patients and assessed for anti-MDA5 Abs. This study was reviewed and approved by the Research Ethics Committees of Tokyo Hospital (190010) and Sagamihara Hospital and the Central Institutional Review Board of the National Hospital Organization. Written informed consent was obtained from all patients. This study was conducted in accordance with the principles expressed in the Declaration of Helsinki. 

### 2.2. Detection of Anti-MDA5 Abs

Anti-MDA5 Abs were detected using Mesacup anti-MDA5 tests, according to the manufacturer’s instructions (Medical & Biological Laboratories, Tokyo, Japan, User’s manual, https://www.info.pmda.go.jp/downfiles/ivd/PDF/130249_22700EZX00013000_A_01_01.pdf, accessed on 20 January 2023). Sera were diluted 1:100 with the dilution buffer of the kit. An index value was calculated according to the manufacturer’s instructions as follows: index value = (optical density value of sample—optical density value of blank)/(optical density value of positive control—optical density value of blank) × 100. The cut-off value was set to 8.156, based on the 98th percentile among 52 healthy controls (mean age ± SDM: 35.4 ± 11.1, male number: 2 [3.8%]). RF was also measured with an N-latex RF kit (Siemens Healthcare Diagnostics, München, Germany), which measured IgM class RFs; the cut-off value was 15 U/mL. ACPA IgG was detected with Mesacup-2 test CCP; the cut-off value was 4.5 U/mL. KL-6 was measured with the Picolumi KL-6 Electrochemiluminescence immunoassay system (EIDIA Co., Ltd., Tokyo, Japan); the cut-off value was 500 U/mL. SP-D was measured with SP-D Yamasa EIA II kits (Yamasa Corporation, Choshi, Japan); the cut-off value was 110 ng/mL. The results of RF, ACPA, KL-6, and SP-D for some of the RA patients have been reported previously [10]. Steinbrocker stages were classification criteria of RA progression stages from I to IV and were evaluated as previously described [24].

### 2.3. Statistical Analysis 

The clinical characteristics of the subsets of RA patients were compared with RA patients without CLD by Mann–Whitney U tests or Fisher’s exact tests. The presence of Abs was compared in RA patients without CLD by Mann–Whitney U tests or Fisher’s exact tests. Multiple logistic regression analysis was conducted to create an MDA5-index with covariates with *p* _adjusted_ < 0.1 (anti-MDA5 Abs, age [years], Steinbrocker stage [1,2,3,4], and smoking status [current smoker: 2, past smoker: 1, never smoker: 0]). ROC curves for Abs were used to compare RA patients with or without CLD. Area under the curve (AUC) values for ROC curves with 95% confidence intervals (CI) were calculated and compared with the AUC value of 0.5 or other ROC curves by Chi-square analysis. The optimized cut-off levels based on the highest Youden’s index were estimated.

## 3. Results

### 3.1. Clinical Manifestations of Patients with RA 

The clinical manifestations of the RA patients investigated here are described in Table 1 and Table 2. The mean age, male:female ratio, age at onset, percentage of smokers or past smokers, KL-6 levels and SP-D levels were higher, and the Steinbrocker stage lower, in RA patients with ILD than in those without CLD. The mean age, age at onset, KL-6 levels and SP-D levels were higher in RA patients with ADs. The mean age, male:female ratio, age at onset, percentage of smokers or past smokers, KL-6 levels and SP-D levels were higher, and the Steinbrocker stage lower, in RA patients with emphysema. 

### 3.2. Presence of Anti-MDA5 Abs in RA Patients

Anti-MDA5 Abs were quantified in the sera of RA patients, with the results shown in Table 1 and Table 2. Anti-MDA5 Ab levels were significantly associated with ADs (mean ± SDM, 4.4 ± 2.4 vs. 4.0 ± 4.2, *p =* 0.0001), emphysema (4.1 ± 1.9 vs. 4.0 ± 4.2, *p =* 0.0273) and CLD (4.4 ± 3.3 vs. 4.0 ± 4.2, *p =* 0.0018). RF and ACPA were also quantified in RA patient sera (Table 2). RF levels were associated with ILD (475.5 ± 1124.6 vs. 262.7 ± 609.5 [U/mL], *p =* 0.0020), emphysema (835.1 ± 1947.1 vs. 262.7 ± 609.5 [U/mL], *p =* 0.0007), and CLD (387.2 ± 1010.0 vs. 262.7 ± 609.5 [U/mL], *p =* 0.0018). ACPA levels were associated with RA in patients with emphysema (433.1 ± 393.8 vs. 275.3 ± 306.2 [U/mL], *p =* 0.0052). Assessments of positivity for anti-MDA5 Abs, RF, and ACPA were also conducted in the RA patients (Supplementary Table S1). Although similar tendencies were observed, no significant associations were detected. Anti-MDA5 Ab levels in RA were also compared with those in healthy controls (Supplementary Tables S2 and S3) and were higher than the controls. Thus, anti-MDA5 Ab titers were associated with ADs and CLD in RA but not with RA in general. 

**Figure 1:**
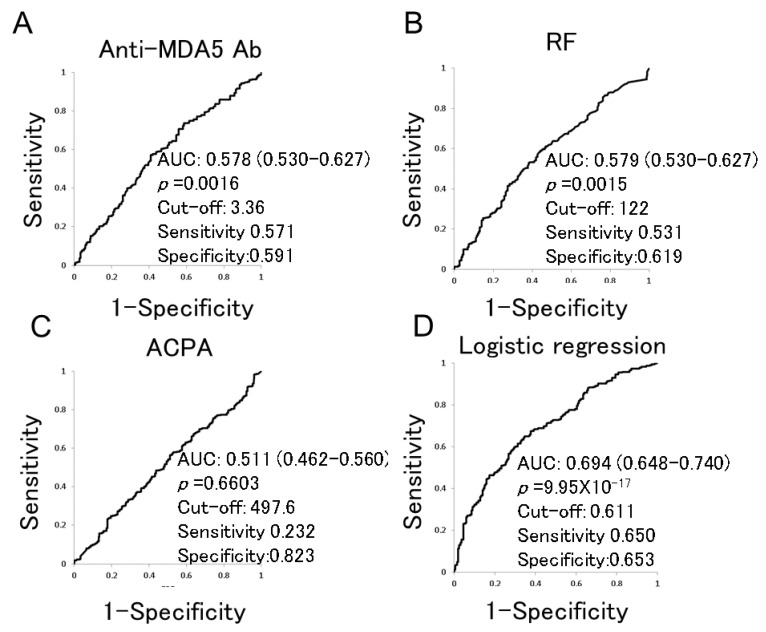
Receiver operating characteristic (ROC) curves using anti-MDA5 Abs (**A**), RF (**B**), ACPA (**C**), and multiple logistic regression analysis (**D**) for comparisons between CLD(+) and CLD(−) RA.

The ROC curve for anti-MDA5 Abs was compared in RA patients with and without CLD (Figure 1A). The AUC value of the ROC curves for anti-MDA5 Abs (0.578, 95% CI 0.530−0.627) was similar to RF (*p =* 0.9411, Figure 1B) but tended to be higher than ACPA (*p =* 0.0665, Figure 1C). Thus, anti-MDA5 Ab values have similar characteristics to RF for the diagnosis of CLD.

ROC curves for anti-MDA5 Abs (A), RF (B), ACPA (C) and multiple logistic regression analysis with anti-MDA5 Abs, age (years), Steinbrocker stage (1–4), and smoking status (current smoker: 2, past smoker: 1, never smoker: 0) (D) were generated to compare CLD(+) and CLD(−) RA. The area under the curve (AUC) values of the ROC curves with 95% confidence intervals and the optimized cut-off levels with specificities and sensitivities are shown: MDA5: melanoma differentiation-associated gene 5, Ab: antibody, RF: rheumatoid factor, ACPA: anti-cyclic citrullinated peptide antibody, ROC: receiver operating characteristic, AUC: area under the curve, and CLD: chronic lung disease.

The results of multiple logistic regression analysis of anti-MDA5 Abs and patients’ clinical characteristics are shown in Table 3. From these data, anti-MDA5 Abs, age, Steinbrocker stage, and smoking status were selected (*p*_adjusted_ < 0.1) to create an MDA5-index defined as: 0.0636 × (anti-MDA5 Abs) + 0.0554 × (age) − 0.2037 × (Steinbrocker stage) + 0.4615 × (smoking status) − 3.1211. The ROC curve AUC value was 0.694 (95% CI 0.648–0.740, *p =* 9.95 × 10^−17^, Figure 1D), which was higher than for anti-MDA5 Abs (*p =* 5.08 × 10^−5^) or RF (*p =* 0.0010). Thus, multiple logistic regression analysis using anti-MDA5 Abs and certain clinical characteristics resulted in the generation of an MDA5-index with the highest AUC value.

## 4. Discussion

In this study, anti-MDA5 Abs were found to be associated with ADs in RA patients. The AUC values of the ROC curves for anti-MDA5 Abs and RF were similar when comparing RA with and without CLD. An MDA5-index was generated from anti-MDA5 Abs, age, Steinbrocker stage, and smoking status with a ROC curve AUC value higher than for anti-MDA5 Abs or RF alone.

An association of RF with ILD has been previously reported in RA [12,13] and was confirmed in the present study. The association of ACPA with ILD was also reported in RA [12,14,15], but this was not confirmed here. On the other hand, we found that anti-MDA5 Abs were associated with ADs in RA, leading to the notion that anti-MDA5 Abs may be involved in the pathogenesis of ADs. In contrast, anti-MDA5 Abs, RF and ACPA were found to be associated with emphysema (but a possible confounding effect of smoking status could not be excluded). Thus, different specific roles of anti-MDA5 Abs, RF, and ACPA in the pathogenesis of CLD in RA patients should be investigated.

It was found that some clinical characteristics were associated with CLD in RA, though a causal relationship could not be confirmed in this study. These might be confounding factors. Using multiple logistic regression analyses of anti-MDA5 Abs and the clinical characteristics, we created an MDA5-index. This suggested that anti-MDA5 Abs could be used to generate a composite biomarker for CLD in RA. The cut-off level set in this study for anti-MDA5 Ab positivity (8.156) was lower than the kit manufacturer’s recommended cut-off level (32) for clinically amyopathic dermatomyositis developing into rapidly progressive ILD. Anti-MDA5 Ab index levels >32 was observed in one RA patient without CLD in the present study. These data suggest that the characteristics of anti-MDA5 Abs regarding ADs in RA patients are different from clinically amyopathic dermatomyositis developing into rapidly progressive ILD. Thus, anti-MDA5 Abs could be used as an alternative biomarker for ADs or CLD in RA. However, results from anti-MDA5 Abs, RF and ACPA indicated that they are not better biomarkers for ILD in RA than KL-6 or SP-D. 

Anti-MDA5 Abs have been detected in RA or idiopathic interstitial pneumonia patients developing rapidly progressive ILD [25,26,27]. They might also be detectable in ADs patients without RA. It was reported that pharmacological Janus kinase inhibition is effective against rapidly progressive ILD in dermatomyositis patients with anti-MDA5 Abs [28], suggesting that these drugs may also be useful for controlling ADs in RA patients. The titer of anti-MDA5 Abs was influenced by the treatment for ILD complicated with dermatomyositis [29] and the results of anti-MDA5 Ab levels in this study would be modified by the treatment for RA or RA disease activities.

To the best of our knowledge, this is the first report on anti-MDA5 Ab profiles in RA patents with CLD, describing an association of anti-MDA5 Abs with ADs. The independent association of anti-MDA5 Ab levels with CLD in RA was not confirmed in logistic regression analysis after adjustment. Because the study sample size was modest, larger-scale studies on anti-MDA5 Abs in RA should be performed to validate these results. The anti-MDA5 Ab profiles in patients with collagen vascular diseases other than RA or dermatomyositis should also be analyzed in future studies. The associations of anti-MDA5 Abs in other ethnic populations should be analyzed, since this study was performed only in Japanese populations. Anti-MDA5 Ab levels in RA should be compared with age-matched healthy controls, because age-matched controls were not available in this study.

## Figures and Tables

**Table 1:** Characteristics and anti-MDA5 Ab of RA patients.

	ILD		UIP		NSIP		ADs		Emphysema	
		*p*		*p*		*p*		*p*		*p*
Number	138		63		75		166		39	
Mean age, years (SD)	68.6 (9.1)	4.48 × 10^−7^	70.0 (10.0)	2.46 × 10^−6^	67.5 (8.1)	0.0009	67.9 (10.5)	4.54 × 10^−6^	66.8 (8.2)	0.0301
Male, *n* (%)	37 (26.8)	* 0.0307	23 (36.5)	* 0.0014	14 (18.7)	* 0.7239	28 (16.9)	* 1.0000	24 (61.5)	* 2.68 × 10^−8^
Age at onset, years (SD)	56.5 (14.0)	9.64 × 10^−7^	58.0 (15.7)	2.66 × 10^−5^	55.2 (12.5)	0.0004	54.4 (15.5)	5.87 × 10^−5^	57.4 (11.7)	0.0001
Steinbrocker stage III and IV, *n* (%)	58 (42.6)	* 0.0060	29 (47.5)	* 0.1473	29 (38.7)	* 0.0046	84 (53.2)	* 0.3444	13 (33.3)	* 0.0051
Smoker or past smoker, *n* (%)	56 (43.1)	* 0.0063	28 (47.5)	* 0.0072	28 (39.4)	* 0.1008	55 (37.4)	* 0.0816	30 (85.7)	* 1.37 × 10^−10^
KL-6, U/mL (SD)	822.3 (776.2)	<1 × 10^−16^	904.7 (849.2)	1.04 × 10^−14^	748.6 (703.0)	5.88 × 10^−14^	370.3 (300.5)	0.0002	570.8 (455.3)	4.23 × 10^−7^
SP-D, ng/mL (SD)	138.2 (152.2)	2.82 × 10^−13^	149.6 (105.5)	1.41 × 10^−11^	127.4 (186.4)	1.67 × 10^−7^	78.7 (78.3)	0.0072	94.4 (68.4)	6.84 × 10^−5^
Anti-MDA5 Ab, index value (SD)	4.4 (4.4)	0.4479	4.7 (4.6)	0.1289	4.2 (4.2)	0.8204	4.4 (2.4)	0.0001	4.1 (1.9)	0.0273
RF, U/mL (SD)	475.5 (1124.6)	0.0020	454.9 (888.5)	0.0032	492.9 (1295.9)	0.0472	208.0 (324.1)	0.1693	835.1 (1947.1)	0.0007
ACPA, U/mL (SD)	339.6 (714.2)	0.8122	260.7 (273.2)	0.8615	403.8 (927.8)	0.8428	271.0 (346.6)	0.5776	433.1 (393.8)	0.0052

RA: rheumatoid arthritis, ILD: including interstitial lung disease, UIP: usual interstitial pneumonia, NSIP: nonspecific interstitial pneumonia, ADs: airway diseases, CLD: chronic lung disease, MDA5: melanoma differentiation-associated gene 5, Ab: antibody, RF: rheumatoid factor, ACPA: anti-citrullinated peptide antibody. ILD group includes UIP and NSIP groups. Data are presented as the mean value or number of each group. Statistical differences were tested in comparison with the CLD(−) population by Fisher’s exact test using 2 × 2 contingency tables or the Mann–Whitney U test. * Fisher’s exact test was employed.

**Table 2:** Characteristics and anti-MDA5 Ab in RA patients with or without CLD.

	CLD(+)		CLD(−)
		*p*	
Number	343		215
Mean age, years (SD)	68.1 (9.7)	1.36 × 10^−8^	62.4 (11.1)
Male, *n* (%)	89 (25.9)	* 0.0122	36 (16.7)
Age at onset, years (SD)	55.6 (14.5)	1.35 × 10^−8^	48.6 (13.5)
Steinbrocker stage III and IV, *n* (%)	155 (46.5)	* 0.0087	125 (58.1)
Smoker or past smoker, n (%)	141 (45.2)	* 0.0001	57 (28.2)
KL-6, U/mL (SD)	601.6 (619.7)	1.33 × 10^−15^	283.3 (274.3)
SP-D, ng/mL (SD)	109.5 (123.7)	2.09 × 10^−10^	49.9 (39.4)
Anti-MDA5 Ab, index value (SD)	4.4 (3.3)		4.0 (4.2)
RF, U/mL (SD)	387.2 (1010.0)	0.0018	262.7 (609.5)
ACPA, U/mL (SD)	316.2 (530.1)	0.6626	275.3 (306.2)

RA: rheumatoid arthritis, ILD: interstitial lung disease, UIP: usual interstitial pneumonia, NSIP: nonspecific interstitial pneumonia, ADs: airway diseases, CLD: chronic lung disease, MDA5: melanoma differentiation-associated gene 5, Ab: antibody, RF: rheumatoid factor, ACPA: anti-citrullinated peptide antibody. ILD group includes UIP and NSIP groups. CLD(+) group includes UIP, NSIP, ADs, and emphysema groups. Data are presented as the mean value or number of each group. Statistical differences were tested in comparison with the CLD(−) population by Fisher’s exact test using 2 × 2 contingency tables or the Mann–Whitney U test. * Fisher’s exact test was employed.

**Table 3:** Multiple logistic regression analysis of Abs and clinical manifestations for RA with CLD.

	Unconditioned			Conditioned on the Other Factors		
Clinical Manifestations	OR	95%CI	*p*	OR_adjusted_	95%CI	*p*_adjusted_
Anti-MDA5 Ab (index value)	1.0309	(0.9782~1.0865)	0.2559	1.0608	(0.9919~1.1345)	0.0851
RF(IU/mL)	1.0002	(0.9999~1.0005)	0.1220	1.0002	(0.9999~1.0005)	0.2113
ACPA (U/mL)	1.0002	(0.9998~1.0007)	0.3153	1.0001	(0.9997~1.0006)	0.5674
Age, years	1.0542	(1.0357~1.0730)	4.88 × 10^−9^	1.0592	(1.0298~1.0895)	6.24 × 10^−5^
Male	1.7422	(1.1313~2.6831)	0.0117	1.2959	(0.7761~2.1639)	0.3217
Age at onset, years	1.0351	(1.0221~1.0482)	7.95 × 10^−8^	0.9967	(0.9744~1.0195)	0.7758
Steinbrocker stage	0.7899	(0.6843~0.9117)	0.0013	0.8094	(0.6602~0.9924)	0.0420
Smoking status	1.5497	(1.1993~2.0025)	0.0008	1.5308	(1.1642~2.0129)	0.0023

RA: rheumatoid arthritis, CLD: chronic lung diseases, MDA5: melanoma differentiation-associated gene 5, Ab: antibody, RF: rheumatoid factor, ACPA: anti-citrullinated peptide antibody, OR: Odds ratio, CI: confidence interval. *p*, OR, 95%CI, *p*_adjusted_, OR_adjusted_ were calculated by logistic regression analysis on RA patients. Smoking status of RA patients were 0: never smoker, 1: past smoker, and 2: current smoker.

## Data Availability

The data that support the findings of this study are not publicly available due to privacy and ethical restrictions. The data are available from the corresponding author upon reasonable request.

## Supplementary Materials

The following supporting information can be downloaded at: https://www.mdpi.com/article/10.3390/medicina59020363/s1, Figure S1: Distribution of anti-MDA5 Abs in 52 healthy controls. Table S1: The positivity of RF, ACPA, and anti-MDA5 Ab in the RA patients. Table S2: The comparison of anti-MDA5 Ab in the RA patients and controls.
