# Supplementary material for: Antibodies against Serum Anti-Melanoma Differentiation-Associated Gene 5 in Rheumatoid Arthritis Patients with Chronic Lung Diseases
Source: Medicina (Kaunas). 2023 Feb 14;59(2):363. doi: 10.3390/medicina59020363 (PMC9962840; doi:10.3390/medicina59020363)
Supplement: Supplementary file 1 [file medicina-59-00363-s001.zip › medicina-2202368-supplementary.pdf]

Supplementary Materials: Antibodies against Serum Anti-Melanoma Differentiation-Associated Gene 5 in Rheumatoid Arthritis Patients with Chronic Lung Diseases

Supplementary Table S1. The positivity of RF, ACPA, and anti-MDA5 Ab in the RA patients.

	ILD		UIP		NSIP		AD		Emphysema		CLD(+)		CLD(−)
		P		*P*		*P*		*P*		*P*		*P*	
Anti-MDA5 Ab positive, n (%)	12 (8.7)	0.1056	6 (9.5)	0.1147	6 (8.0)	0.2272	12 (7.2)	0.2578	2 (5.1)	0.6788	26 (7.6)	0.1502	9 (4.2)
RF positive, n (%)	126 (91.3)	0.0532	57 (90.5)	0.2276	69 (92.0)	0.0851	146 (88.0)	0.3035	34 (87.2)	0.8111	306 (89.2)	0.0692	180 (83.7)
ACPA positive, n (%)	127 (92.0)	0.8406	59 (93.7)	1.0000	68 (90.7)	0.6223	147 (88.6)	0.2115	36 (92.3)	1.0000	310 (90.4)	0.4433	199 (92.6)

RA: rheumatoid arthritis, ILD: interstitial lung disease, UIP: usual interstitial pneumonia, NSIP: nonspecific interstitial pneumonia, AD: airway disease, CLD: chronic lung disease, RF: rheumatoid factor, ACPA: anti-citrullinated peptide antibody, MDA5: melanoma differentiation-associated gene 5. ILD group includes UIP and NSIP groups. CLD(+) group includes UIP, NSIP, AD, and emphysema groups. Number of each group is shown. Percentages are shown in parenthesis. Difference was tested in the comparison with the CLD(−) population by Fisher's exact test using 2 × 2 contingency tables.

Supplementary Table S2. The comparison of anti-MDA5 Ab in the RA patients and controls.

	ILD	UIP	NSIP	AD	Emphysema	CLD(+)	CLD(−)	RA	Controls
	P	*P*	*P*	*P*	*P*	*P*	*P*	*P*	
Anti-MDA5 Ab	6.31×10^{-5}	7.33×10^{-5}	0.0015	2.70×10^{-9}	8.42×10^{-7}	1.70×10^{-8}	0.0002	3.26×10^{-7}	2.9 (2.2)

RA: rheumatoid arthritis, ILD: interstitial lung disease, UIP: usual interstitial pneumonia, NSIP: nonspecific interstitial pneumonia, CLD: chronic lung disease, MDA5: melanoma differentiation-associated gene 5. ILD group includes UIP and NSIP groups. CLD(+) group includes UIP, NSIP, AD, and emphysema groups. Number of controls is shown. Percentages are shown in parenthesis. Difference was tested in the comparison with the control population by Mann–Whitney U test.

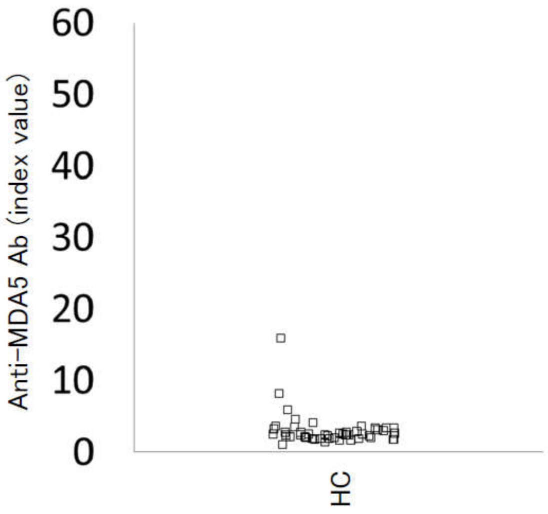

Supplementary Figure S1. Distribution of anti-MDA5 Abs in 52 healthy controls. HC: healthy controls, MDA5: melanoma differentiation-associated gene 5, Ab: antibody.
